# Handbook of Materia Medica for Trained Nurses, Including Sections on Therapeutics and Toxicology, and a Glossary of Terms, with Dose and Use of Each Drug

**Published:** 1898-12

**Authors:** 


HANDBOOK OF MATERIA MEDICA for Trained Nurses, including sections on Therapeutics and Toxicology, and a Glossary of Terms, with Dose and Use of Each Drug. By JOHN E. GROFF, Ph. G., Apothecary in the Rhode Island Hospital, Providence. 12mo, pp. 243. Philadelphia: P. Blakiston's Son & Co. 1898.

The author has prepared a work for nurses which will be found of value by them, both in and out of the training school. It covers, in a brief and simple way, the entire field of materia medica, including weights and measures and the sources, preparations, dosage and the action of the principal officinal remedies. A chapter on toxicology is added which gives in case of the common poisons the prominent symptoms as distinguished from the symptoms of disease. The book concludes with a glossary, containing a complete list of all the official drugs with the Latin and English name, medicinal use and dose of each. Each chapter is supplemented by a list of questions. One disposed to be critical might ask why convalluria is classed as a cardiac depressant, but on the whole the book is excellent.

